# Enhancing Intermediate Vision in Patients Affected by Epiretinal Membrane Treated by Phaco-Vitrectomy

**DOI:** 10.3390/jcm12155016

**Published:** 2023-07-30

**Authors:** Fabrizio Giansanti, Francesco Dragotto, Cristina Nicolosi, Ludovica Alonzo, Lorenzo Cifarelli, Fabrizio Gaetano Saverio Franco, Lorenzo Vannozzi, Giacomo Abbruzzese, Daniela Bacherini, Gianni Virgili

**Affiliations:** Department of Neurosciences, Psychology, Drug Research and Child Health, Eye Clinic, University of Florence, AOU Careggi, 50121 Florence, Italy; fabrizio.giansanti@unifi.it (F.G.); cristina.nicolosi@unifi.it (C.N.); ludovica.alonzo@unifi.it (L.A.); lorenzo.cifarelli@unifi.it (L.C.); fabriziogsfranco@gmail.com (F.G.S.F.); lorenzo.vannozzi@tin.it (L.V.); abgiacomo@gmail.com (G.A.); daniela.bacherini@gmail.com (D.B.); gianni.virgili@unifi.it (G.V.)

**Keywords:** enhanced monofocal, IOL, ERM, cataract, presbyopia correction

## Abstract

Background: The aim of this research was to see if a refractive enhanced monofocal IOL (Eyhance IOL, IOL Abbott Medical Optics, Inc., Santa Ana, CA, USA) can provide better intermediate vision in patients undergoing phaco-vitrectomy due to cataract and epiretinal macular membrane (ERM). Methods: A nonrandomized prospective observational comparative study enrolled patients affected by cataract and ERM undergoing phaco-vitrectomy. A follow up of 6 months was established. Corrected and uncorrected visual acuity of both monocular and binocular types were assessed regarding intermediate and far distances. The CATQUEST 9-SF questionnaire was administered preoperatively and at the last follow-up. Results: Twenty-three eyes of twenty-three patients were enrolled, with 11 in the enhanced monofocal group. The uncorrected and corrected distance visual acuity after 6 months was not statistically different. Both monocular and binocular uncorrected intermediate visual acuity after 6 months were higher in the enhanced monofocal group (*p* < 0.001). The corrected intermediate visual acuity after 6 months was higher in the enhanced monofocal group (*p* = 0.01). The CATQUEST-9SF questionnaire showed significant differences in the variation between the preoperative condition and six-month postoperative results (*p* < 0.001). Conclusions: This refractive enhanced monofocal IOL can provide better intermediate vision compared to a standard monofocal IOL in patients undergoing phaco-vitrectomy due to cataracts and ERM. Further studies are necessary to confirm these results.

## 1. Introduction

The goal of each ophthalmic surgery is to restore the normal functionality of the eye structures in order to provide the best functional recovery from the treated disease. In patients affected by cataract and epiretinal macular membrane (ERM), two different problems have to be solved, and phacoemulsification with intraocular lens (IOL) implantation combined with pars plana vitrectomy (PPV) and membrane peeling is the treatment normally proposed [1].

Features and symptoms of ERM are variable, and initial observation can be chosen to avoid unnecessary surgery until clinical or ultrasrtuctural progression is overcome [2].

However, postoperative visual function is related to preoperative clinical conditions, so early surgery is frequently advocated [3]. These results have been achieved thanks to technical progression of the surgical maneuvers that have reduced drastically the postoperative morbidity and the recurrence rate. Specifically, ILM removal seems to reduce the recurrence [4]. Regarding safety and better outcomes, lower gauge surgery and dye-assisted peeling have changed the approach on this kind of surgery [5].

Cataract surgery has also changed and improved over time, permitting patients to achieve better postoperative outcomes. Specifically, cataract refractive surgery also nowadays has the aim to satisfy the increasing need for vision improvement at intermediate distance in modern everyday life [6,7,8], overcoming the presbyopia problem and spectacle independence. One solution is represented by the implant of multifocal IOLs, which are currently increasingly implanted after cataract surgery [6,7], even if these premium IOLs have as principal side effects a possible reduction of contrast sensitivity and dysphotopsia phenomena, mainly glare and halos [8,9]. Another option is represented by enhanced depth of focus IOLs and enhanced monofocal lenses [10,11], which provide a high spectacle independence [12] mostly in the intermediate vision, without compromising contrast sensitivity [10].

The increasing need for vision improvement at intermediate distance in everyday life [13] can be extended also to patients undergoing combined phacovitrectomy for ERMs, which are usually considered unsuitable for the implant of multifocal IOLs. Macular disorders indeed have been generally considered a relative contraindication for the implantation of premium multifocal IOLs because of the possible combined effect of the retinal pathology and the IOL characteristics on reducing contrast sensitivity [14]. Furthermore, nowadays, evidence-based guidelines are not available for the implantation of multifocal IOLs in eyes affected by retinal pathologies, and clinical outcomes have not been adequately studied yet. The employment of enhanced monofocal IOLs, which are supposed to increase intermediate vision without compromising the contrast sensitivity [10], have not been investigated yet in this category of patients.

In the hypothesis that this type of IOL may represent a possible option of presbyopia correction in ERM patients, undergoing phaco vitrectomy, we conducted the present study with the aim of assessing the clinical outcomes of the implantation of enhanced monofocal IOLs in this category of patients, in comparison to standard monofocal IOLs.

## 2. Materials and Methods

This nonrandomized prospective observational comparative study involved patients affected by ERM and cataract, with of less than 0.75 diopters (D) astigmatism, scheduled for phaco-vitrectomy in the ophthalmology service of Careggi University Hospital, Italy. We compared patients who underwent phaco-vitrectomy for ERM between January 2022 and May 2022 receiving either a monofocal IOL or an enhanced monofocal IOL.

Sample size calculation was conducted using the ‘power two means’ command in Stata 17.0 (StataCorp, College Station, TX, USA). Based on previous studies conducted by our group, the SD of the final UCVA was assumed to be 0.1 logMAR in the two groups. We considered that a difference of 0.1 logMAR (one Snellen line) was a minimal important difference between groups; 0.1 logMAR is also the coefficient of reproducibility of a standard ETDRS chart in subjects with normal vision. Based on these assumptions, we calculated that two equal groups of 12 subjects each were allowed to yield 80% power to detect a difference of 0.1 logMAR between treatment arms, with alpha set at 0.05.

Patients were excluded if one of the following conditions was found: amblyopia, previous ocular surgery including corneal transplant, previous vitrectomy, corneal refractive surgery, scleral buckle surgery, uveitis—both recurrent or chronic, diabetic retinopathy, glaucoma, keratoconus, and corneal endothelial dystrophy. Patients affected by pre-existing ocular and retinal diseases other than ERM were excluded.

This study followed the tenets of the Declaration of Helsinki, and it was approved by the local institutional review board (n. 21267_oss). Patients were informed about the study, and an informed consent was provided and signed before undergoing clinical examinations and surgery, and one week time to decide whether to participate or not was given; if they refused to participate, phacovitrectomy was performed, implanting a monofocal IOL. Follow up visits scheduled remain the same as for study participants. Any contraindications of implanting the DIB IOL in ERM affected patients previously, during or after vitrectomy was not found in the technical product sheet. Participants were informed to promptly refer if ocular pain, blurred vision, or any other ocular symptoms occurred, wherein an ophthalmology check was provided within the 24 subsequent hours.

### 2.1. Preoperative and Postoperative Patient Evaluation

Subjective refraction was obtained using the duochrome test to determine the sphere and the astigmatic clock dial test to determine the axis and the cylinder power. Objective refraction was then obtained using the AR-1S, NIDEK Co. Ltd., Gamagori, Japan. Then, 100% contrast with the Early Treatment Diabetic Retinopathy Study (ETDRS) charts were used at 85 cd/m^2^ (photopic condition) and at 4 m distance for the measurement of the uncorrected and corrected distance visual acuity (UDVA) (CDVA). To evaluate, cataract examiners (F.D., F.G.) conducted a dilated anterior segment examination in mydriasis, and cataract was defined as at least NUC-2, COR-2, or PSC-2 (who/pbd standards). ERM was diagnosed by means of fundus ophthalmoscopy first and then confirmed by macular optical coherence tomography (OCT) (RS-3000 NIDEK Co. Ltd., Gamagori, Japan) analysis, and classification was graded following stages proposed by Govetto et al. [15,16]. In addition, every patient underwent an ophthalmological evaluation including optical biometry (IOLMaster 500, Carl Zeiss Meditec AG), bio-microscopy, Goldmann applanation tonometry, and dilated fundoscopy. The MS-39 topo-tomographer (CSO, Scandicci Italy) was used to obtain corneal anterior curvature and simulated keratometry (Sim-K). Biometry data were used to choose IOL power and to predict postoperative refraction; according to the axial length, different calculations were made in order to achieve a postoperative refraction closest to emmetropia (Holladay 1 and Barrett universal II formula for axial lengths > 22.0 mm and <25.0 mm and Hoffer Q, Kane, and Barrett universal II formula for axial lengths ≤ 22.0 mm), and for each patient, an arithmetic mean of all the IOL power given by each formula was calculated and then the IOL power was chosen [17,18,19,20]. A myopic shift after the ERM peeling was considered.

The CATQUEST-9SF (9 item short formula) questionnaire was explained and administered to the patients for the preoperative evaluation.

Two groups of patients were created: the first received a monofocal IOL (Tecnis ZCB00 IOL Abbott Medical Optics, Inc., Santa Ana, CA, USA). It is a single-piece hydrophobic acrylic monofocal IOL with a diameter of 6 mm; its anterior surface is aspheric, and the frosted posterior square edge is frosted and continuous over 360°. The second group was implanted with an enhanced monofocal IOL: the Tecnis Eyhance IOL (DIB00) (Abbott Medical Optics, Inc., Santa Ana, CA, USA). It was chosen to minimize the differences between the two IOLs as it has barely the same features of the ZCB00 IOL, except for an enhanced anterior optic profile with an anterior surface power that continuously increases from the periphery to the center, resulting in a negative spherical aberration [21]. This refractive technology improves vision for intermediate tasks compared with a standard monofocal IOL [21,22]. The two IOLs have no specific macroscopic differentiating features, so it is impossible to the clinician to recognize at the slit lamp examination, which of the two lenses were implanted. Thanks to this, the study was conducted in blinding the post-operative examinator on the IOL model, avoiding further biases.

Post hoc analysis was conducted to evaluate if differences occurred between the two groups regarding demographic data and erm severity.

The mean age in the monofocal group was 75.75 ± 9.52 yo, and it was 74.54 ± 6.58 yo in the monofocal group, without any significant differences between the two groups (*p* > 0.05). In the monofocal group, female participants were 5 and males 7 in total; in the enhaced monofocal group, they were 5 and 6, respectively. No statistically significant differences were found between the two groups (*p* > 0.05). Moreover, for the pre-operative corrected distance visual acuity (mean monofocal group: 0.51 ± 0.26 logMAR and mean enhanced monofocal group 0.41 ± 0.14 logMAR) and for the cylinder (mean monofocal group: 0.47 ± 0.17 D and mean enhanced monofocal group 0.49 ± 0.18 D), the two groups were comparable (*p* > 0.05). The ERM severities (Table 1) were comparable for each stage in the two groups, without significative differences (*p* > 0.05). All the reported data are summarized in Table 1. 

Postoperatively, patients were evaluated at 1 day, 1 week, 1 month, 3 months, and 6 months. At each postoperative visit, in addition to the slit-lamp examination, tonometry, and OCT scan, subjective and objective monocular refraction were obtained at each visit following the same methods as preoperatively. Binocular UDVA, monocular, and binocular uncorrected intermediate visual acuity (UIVA) at 66 cm, and CDVA, distance-corrected intermediate visual acuity (DCIVA) were measured at 85 cd/m^2^. Also, patients’ satisfaction was assessed using again the CATQUEST-9SF questionnaire administered during the 6th month follow-up visit. Intermediate visual acuities were measured by ETDRS printed charts (Precision Vision, Woodstock, IL, USA); a light meter (ST-1300, STANDARD Instruments Co., Ltd., Hong Kong, China) was used to measure the room illumination, and then light was adjusted to obtain photopic conditions. To create defocus, 0.50 D increments in the lenses were consecutively added (range +1.00 to −2.50 D) to the best distance correction, and then visual acuity was tested. This operation was done both for distance and for intermediate visual acuity. Visual performance was also evaluated by measuring the monocular contrast sensitivity using Pelli–Robertson charts under an external illumination of −85 cd/m^2^. 

At the last follow-up visit, the CATQUEST 9-SF was administered to evaluate the vision quality [13]. Questions were explained previously to the patients, before the administration; then, they were left alone to fill in the form.

### 2.2. Surgery

The surgical interventions were conducted under peribulbar anesthesia. After the creation of a superior corneal tunnel incision and a capsulorhexis of about 5.5 mm, the phacoemulsification was performed using the phacovitrector (Constellation, Alcon Laboratories Inc., Fort Worth, TX, USA), and the stop and chop technique was used to fragment the nucleous into pieces; cortical masses were aspirated using a bimanual technique. The IOL was then implanted in the capsular bag, after the injection of a cohesive viscoelastic substance that was then removed carefully. To seal the surgical wounds, periferal hydrosutures were performed in order not to involve the central retina and affect visualization during vitrectomy. At the end of cataract surgery, 1 mg intracameral cefuroxime was injected into the anterior chamber. After that, all eyes underwent a standard sutureless 25-gauge 3-port pars-plana vitrectomy with a wide-angle noncontact viewing system (Resight^®^; Carl Zeiss Meditec AG, Jena, Germany) using the Constellation Vision System (Alcon Laboratories Inc., Fort Worth, TX, USA). Brilliant Blue G (Brilliant Peel^®^, Fluoron GmbH, Ulm, Germany) was used to stain and peel the ERM and the internal limiting membrane using an ILM forceps. The pinch and peel technique was used to elevate the flap at the periphery of the macula. A complete vitrectomy was performed with peripheral indentation, and peripheral retinal photocoagulation was carried out in eyes with peripheral retinal tears or holes. Fluid–air exchange was then performed. At the end of the surgery, 4 mg of betamethasone was injected subconjunctivally.

Each patient followed the same topical therapeutic scheme.

For postoperative prophylaxis against infection, levofloxacin eyedrops 4 times per day for one week were administered [23]. Nonsteroidal anti-inflammatory eyedrops for one month were administered in combination with steroid eyedrops 4 times daily for the first 7 days, then tapered in 3 weeks, in order to manage postoperative anterior and posterior chamber inflammation.

## 3. Results

Twenty-three eyes of twenty-three patients were enrolled into the study between January and May 2022, with 11 in the enhanced monofocal group. One patient was further excluded because he suffered of a rhegmatogenous retinal detachment in the operated eye. Four patients suffered of a postsurgical macular edema, including two in the monofocal group. All the demographic and baseline data are reported in Table 1, and the two groups were comparable.

In Table 2 is a summary of the data collected during the 6 months observational study.

The uncorrected distance visual acuity (UDVA) at 6 month follow-up was found to be not statistically different between the two groups.

The uncorrected intermediate visual acuity (UIVA) at 6 months of follow-up in the two groups was found significantly different between the two groups (*p* < 0.001). The enhanced monofocal IOL group reached a better uncorrected monocular intermediate visual acuity than the standard monofocal IOL group.

The bilateral uncorrected intermediate visual acuity (Bilateral UIVA) at 6 months of follow-up was found to be significantly different between the two groups (*p* < 0.001). The enhanced monofocal IOL group reached a better uncorrected binocular intermediate visual acuity than the standard monofocal IOL group.

The corrected distance visual acuity (CDVA) at 6 months of follow-up was not found to be significantly different between the two groups (*p* > 0.05).

The corrected intermediate visual acuity (CIVA) at 6 months of follow-up in the two groups was found to be significantly different between the two groups (*p* = 0.01). The enhanced monofocal IOL group reached a better corrected monocular intermediate visual acuity than the standard monofocal IOL group.

**Figure 1:**
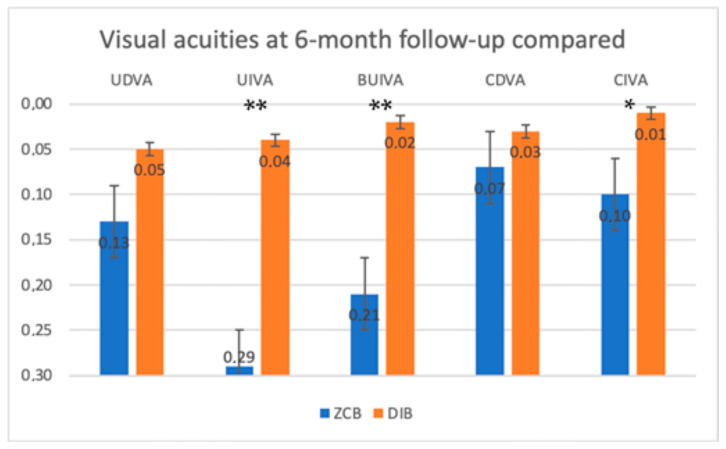
Visual acuities at 6 months of follow up are compared. Asterisk (*) indicates *p* = 0.01, and double asterisks (**) are for *p* < 0.001.

In Figure 1, visual acuities at 6 months of follow up are compared. Asterisks (*) indicate *p* = 0.01, and double asterisks (**) are for *p* < 0.001.

No differences in contrast sensitivity were found between the two groups (*p* > 0.05).

**Figure 2:**
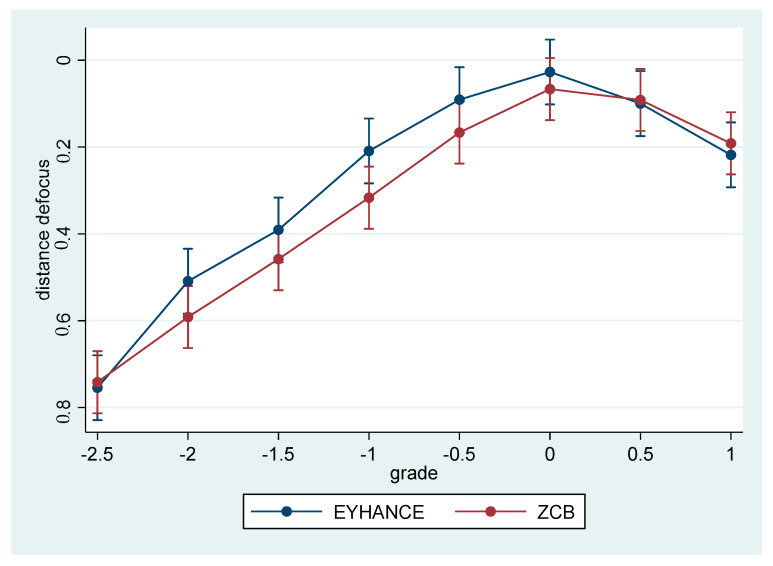
Distance defocus curves.

No differences were found in the overall distance defocus curve between the two groups, even if a smoother profile, in particular in the negative defocus range, seemed to exist (Figure 2).

**Figure 3:**
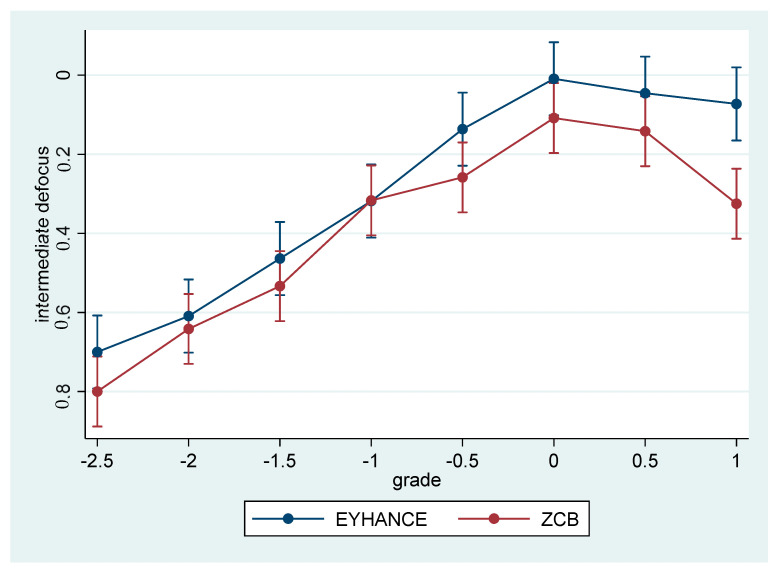
Intermediate defocus curves.

The intermediate defocus curves seemed to differ, showing an overall better performance of the enhanced monofocal lens (Eyhance) (borderline *p*-value = 0.054), in particular for the positive values of defocus (Figure 3).

**Figure 4:**
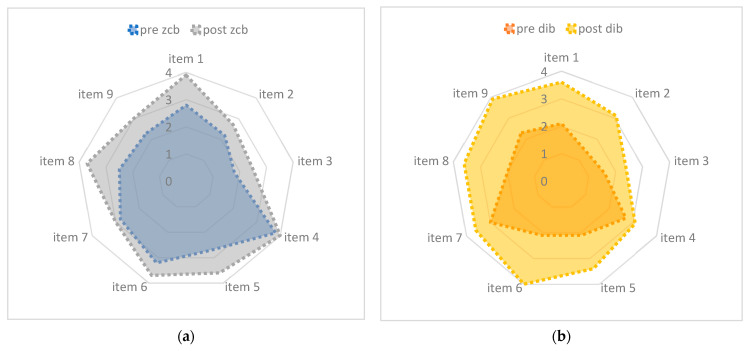
Variation of the CATQUEST-9SF score by item. The difference in the overall amelioration was statistically significant in favor of the Eyhance IOL (*p* < 0.001). (**a**) Variation in the catquest-9sf questionnaire for the monofocal IOL group. (**b**) Variation in the catquest-9sf questionnaire for the enhanced monofocal IOL group.

Regarding the CATQUEST-9SF questionnaire, statistically significant differences were found in the variance between preoperative condition and 6 months of postoperative results (*p* < 0.001) (Figure 4). 

## 4. Discussion

The employment of premium multifocal IOLs in patients affected by retinal diseases, including ERMs, has been generally considered to be not advisable because glare and halos may further reduce the quality of vision, already compromised by the retinal issue [14]. Nevertheless, the greater need for intermediate distance vision improvement in daily routine tasks, with spectacle independence, may be extended also to patients affected by macular disorders, such as ERMs, and we hypothesized that the use of enhanced monofocal IOLs may be useful in this category of patients, without further compromising quality of vision.

Furthermore, with respect to ERMs, it has been recently demonstrated that a surgical treatment in early stages of the disease has better outcomes and recovery than delayed surgery, and early vitrectomy can be considered beneficial in preserving excellent vision [24]. In this perspective, the preservation of a good intermediate vision, without need of spectacles, gains more importance also in patients affected by ERMs, undergoing combined phacoemulsification and vitrectomy, especially in patients with preserved visual acuity. 

Intermediate vision is generally important in the everyday life of both young and elderly people, not only for the large number of reading tasks accomplished (such as smartphone and computer usage) but also for daily routine tasks as descending stairs [25], cooking, and social interaction skills [13]. Notably, leisure and sports also include a variety of activities that require vision at near and intermediate distances [26,27], and so long as the proportion of time spent on these activities ranges from 23% of daily time for individuals aged 55–65 years old to 32% for individuals aged 75 years and older [13], the importance of maintaining good near and intermediate vision after cataract surgery is strengthened. 

Moreover, spectacle independence could allow for the easier completion of the tasks of everyday life [13]. In addition, even for elderly people with reduced mobility, spectacle independence for intermediate tasks could be helpful in moving and recognizing their social activity spaces.

ERM affects patients usually after their sixth decade of life [28,29,30]. These patients are usually socially active and need a range of vision from far to intermediate, but they are considered unsuitable for the implant of multifocal IOLs, due to a possible combined effect of the retinal pathology and the IOL characteristics on reducing contrast sensitivity. 

From this perspective, we conducted the present study to investigate the clinical outcomes of the employment of enhanced monofocal IOLs in patients with ERM undergoing phaco-vitrectomy. These enhanced monofocal IOLs, in fact, are demonstrated to increase the intermediate and near vision, without compromising the contrast sensitivity [10], and thus we hypothesized that they may represent a possible option of presbyopia correction in ERM patients undergoing phaco vitrectomy.

The aim of this study was to investigate if this new kind of IOL can ameliorate the intermediate vision of patients undergoing phaco-vitrectomy for ERM and cataract better than an aspheric monofocal IOL.

We chose to test if the Eyhance platform can be useful in patients affected by ERMs, comparing the performance of an enhanced monofocal IOL (Eyhance, DIB00) to a standard monofocal IOL (ZCB00) in patients undergoing phaco-vitrectomy for ERM. 

We found that the Eyhance model provides higher monocular and binocular UIVA and better results in the CIVA reading at 66 cm at 6 months after surgery, while the final distance visual acuity was not significantly different between the two groups. In addition, the CATQUEST-9SF shows a higher variation regarding the answers between preoperative and six-month postoperative conditions in the enhanced monofocal group.

Previous studies [10,21,31] affirmed that contrast sensitivity in eyes implanted with Eyance IOLs is not poorer than in eyes with ZCB IOLs. This finding is in agreement with our results: in our study, patients receiving Eyhance IOLs did not show a different contrast sensitivity in luminance conditions of 85 cd/m^2^ than patients receiving ZCB IOLs. 

The achievements in intermediate vision are probably possible thanks to a modification of the anterior surface of the lens. A continuous local increase in power is reached thanks to a high order asphericity in the central part of the lens that varies from the center to the periphery. About 85% of the surface is indistinguishable from the surface of the Technis monofocal, enabling both IOLs to provide the same primary corneal spherical aberration correction (−0.27 microns for a 6-mm pupil). Therefore, the refractive IOL design in the Eyhance IOL enables intermediate vision while keeping distance image quality comparable to a standard monofocal aspheric IOL [21].

It must be known that this type of lens is not a multifocal IOL and also not an extended depth of focus (EDOF) IOL, so spectacle independence for near vision is rarely achieved and cannot be proposed.

Moreover, it has been studied [32] that this specific IOL has a high tolerance to decentration, comparable to the ZCB00. Also for this reason, it can be suitable for a combined phacovitrectomy, even if postoperative positioning and gas tamponade could cause a slight movement of the lens.

In our study, Eyhance IOL performed better than ZCB00 IOL regarding corrected and uncorrected monocular intermediate vision (*p* < 0.001 and *p* = 0.01, respectively) and binocular uncorrected intermediate vision (*p* < 0.001). We may speculate that the good performance of this kind of lens in patients who have foveas stretched by ERMs is probably due to a wider range of defocalization of the image on the retina. A standard monofocal lens focalizes light in a specific region by converging light beams and by forming a light cone. The refractive technology of Eyhance IOL stretches the focalization point, transforming it barely into a line [32]. In patients who have foveal architecture altered by ERM, this could be helpful in finding the focalization point. This hypothesis could be supported by the fact that the intermediate defocus curve shows a trend of significance (*p* = 0.054) and can find encouragement in previous studies, showing that Eyhance IOL is more permissive regarding the refractive error [12,21].

The results of our study suggest that Eyhance can be suitable for patients affected by those two pathologies with a corneal astigmatism lower than 0.75 D. Active patients can benefit of intermediate vision in intermediate sight tasks while driving; reading tablets, smartphones, or computers; cooking; and so on. For patients with scarce independence, it has been proven that better seeing can slow the decline progression, especially in dementia-affected patients [33]. Intermediate vision in their life can help in moving around familiar and less familiar places and completing manual tasks. 

The data of the study suggest that patients operated for cataract and ERM with combined phacovitrectomy, receiving an enhanced monofocal IOL, achieve a better intermediate visual acuity without scarifying the distance. Moreover, they are more satisfied with the change from their basal condition, as shown in CATQUEST-9SF outcomes, than patients receiving a standard monofocal IOL. 

To the best of our knowledge, no other studies are reported in the literature regarding the effectiveness of enhanced monofocal IOLs in patients affected by cataracts and ERM. 

This study provides initial encouraging insights into the performance of an enhanced monofocal IOL (Eyhance, DIB00) compared to a standard monofocal IOL (ZCB00) in patients undergoing phaco-vitrectomy for ERM. 

This study has several limitations: first of all, the small sample size does not allow us to have consistent conclusions and subgroup analyses, such as in different stages of the disease, which were unable to have been made.

Also, binocular implantation of the same lens in the contralateral eye could not have been made, as in our series, the pathology had a surgical indication only in one eye for each patient. A prospective interventional study could not have been made, and a double-blinded protocol was not possible. However, in our prospective observational nonrandomized study, the final examiner was blinded in order to avoid biases that derived from knowing the different IOL type, as the two IOLs are visually indistinguishable.

## 5. Conclusions

Eyhance (Abbott Medical Optics, Inc., Santa Ana, CA, USA), a refractive enhanced monofocal IOL, can provide a better intermediate vision compared to a standard monofocal IOL in patients undergoing phaco-vitrectomy due to cataract and ERM. Further studies are necessary to confirm these results.

## Figures and Tables

**Table 1:** Demographic and baseline data. CDVA pre: corrected distance visual acuity at baseline; F/M female patients/male patients; ERM = epiretinal macular membrane.

	Total	Monofocal IOL	Enhaced Monofocal IOL
AGE	75.17 ± 8.08	75.75 ± 9.52	74.54 ± 6.58 (*p* > 0.05)
F/M	10/13	5/7	5/6 (*p* > 0.05)
cylinder	0.48 ± 0.17	0.47 ± 0.17	0.49 ± 0.18 (*p* > 0.05)
CDVA pre (logMAR)	0.46 ± 0.21	0.51 ± 0.26	0.41 ± 0.14 (*p* > 0.05)
staging erm Govetto (*n*)			
1	0	0	0
2	7	3	4 (*p* > 0.05)
3	9	5	4 (*p* > 0.05)
4	7	4	3 (*p* > 0.05)

**Table 2:** The data collected during the 6 months observational study are summarized. Subj, sphere post: subjective spherical component at 6 months; subj. cylinder post: subjective cylindrical component at 6 months; se post: sphere equivalent at 6 months; UDVA 6: uncorrected distance visual acuity at 6 months; UIVA 6: uncorrected intermediate visual acuity at 6 months; BUIVA 6: binocular uncorrected visual acuity at 6 months; CDVA 6: corrected distance visual acuity at 6 months; CIVA 6: corrected intermediate visual acuity at 6 months.

	Total	Monofocal IOL	Enhaced Monofocal IOL	*p*-Value
Subj. sphere post (D)	−0.14 ± 0.26	−0.21 ± 0.28	−0.07 ± 0.22	>0.05
Subj. cylinder post (D)	−0.16 ± 0.74	0.01 ± 0.77	−0.34 ± 0.69	>0.05
se post (D)	−0.22 ± 0.44	−0.21 ± 0.5	−0.23 ± 0.39	>0.05
UDVA 6 (logMAR)	0.1 ± 0.16	0.13 ± 0.18	0.05 ± 0.13	>0.05
UIVA 6 (logMAR)	0.17 ± 0.16	0.29 ± 0.12	0.04 ± 0.08	<0.001 *
BUIVA 6 (logMAR)	0.12 ± 0.15	0.21 ± 0.14	0.02 ± 0.06	<0.001 *
CDVA 6 (logMAR)	0.05 ± 0.11	0.07 ± 0.12	0.03 ± 0.09	>0.05
CIVA 6 (logMAR)	0.06 ± 0.10	0.1 ± 0.13	0.01 ± 0.03	0.01 *

* for statistically significant.

## Data Availability

The data presented in this study are available on request from the corresponding author. The data are not publicly available due to patients’ privacy reasons.

## References

[1] Semeraro F., Morescalchi F., Duse S., Gambicorti E., Russo A., Costagliola C. (2015). Current Trends about Inner Limiting Membrane Peeling in Surgery for Epiretinal Membranes. J. Ophthalmol..

[2] Kofod M., Christensen U.C., La Cour M. (2016). Deferral of surgery for epiretinal membranes: Is it safe? Results of a randomised controlled trial. Br. J. Ophthalmol..

[3] Chua P.Y., Sandinha M.T., Steel D.H. (2022). Idiopathic epiretinal membrane: Progression and timing of surgery. Eye.

[4] Shimada H., Nakashizuka H., Hattori T., Mori R., Mizutani Y., Yuzawa M. (2009). Double staining with brilliant blue G and double peeling for epiretinal membranes. Ophthalmology.

[5] Manousaridis K., Peter S., Mennel S. (2016). 20 g PPV with indocyanine green-assisted ILM peeling versus 23 g PPV with brilliant blue G-assisted ILM peeling for epiretinal membrane. Int. Ophthalmol..

[6] Sieburth R., Chen M. (2019). Intraocular lens correction of presbyopia. Taiwan J. Ophthalmol..

[7] Rho C.R., Kim J.H., Chung I.K., Kim E.C., Han Y.K., Han S.Y., Eom Y., Chung T.Y., Lee D.H., Rho C.R. (2021). Cataract Surgery Practice in the Republic of Korea: A Survey of the Korean Society of Cataract and Refractive Surgery 2020. Korean J. Ophthalmol..

[8] Rosen E., Alió J.L., Dick H.B., Dell S., Slade S. (2016). Efficacy and safety of multifocal intraocular lenses following cataract and refractive lens exchange: Metaanalysis of peer-reviewed publications. J. Cataract Refract. Surg..

[9] Wang S.Y., Stem M.S., Oren G., Shtein R., Lichter P.R. (2017). Patient-centered and visual quality outcomes of premium cataract surgery: A systematic review. Eur. J. Ophthalmol..

[10] Wan K.H., Au A.C.K., Kua W.N., Ng A.L.K., Cheng G.P.M., Lam N.M., Chow V.W.S. (2022). Enhanced Monofocal Versus Conventional Monofocal Intraocular Lens in Cataract Surgery: A Meta-analysis. J. Refract. Surg..

[11] Kohnen T., Suryakumar R. (2020). Extended depth-of-focus technology in intraocular lenses. J. Cataract Refract. Surg..

[12] Sabur H., Unsal U. (2022). Visual outcomes of non-diffractive extended-depth-of-focus and enhanced monofocal intraocular lenses: A case-control study. Eur. J. Ophthalmol..

[13] Ribeiro F., Cochener B., Kohnen T., Mencucci R., Katz G., Lundstrom M., Casanovas A.S., Hewlett D. (2020). Definition and clinical relevance of the concept of functional vision in cataract surgery ESCRS Position Statement on Intermediate Vision: ESCRS Functional Vision Working Group. J. Cataract Refract. Surg..

[14] Braga-Mele R., Chang D., Dewey S., Foster G., Henderson B.A., Hill W., Hoffman R., Little B., Mamalis N., Oetting T. (2014). Multifocal intraocular lenses: Relative indications and contraindications for implantation. J. Cataract. Refract. Surg..

[15] Govetto A., Virgili G., Rodriguez F.J., Figueroa M.S., Sarraf D., Hubschman J.P. (2019). Functional and anatomical significance of the ectopic inner foveal layers in eyes with idiopathic epiretinal membranes: Surgical results at 12 months. Retina.

[16] Govetto A., Lalane R.A., Sarraf D., Figueroa M.S., Hubschman J.P. (2017). Insights Into Epiretinal Membranes: Presence of Ectopic Inner Foveal Layers and a New Optical Coherence Tomography Staging Scheme. Am. J. Ophthalmol..

[17] Holladay J.T., Musgrove K.H., Prager T.C., Lewis J.W., Chandler T.Y., Ruiz R.S. (1988). A three-part system for refining intraocular lens power calculations. J. Cataract Refract. Surg..

[18] Hoffer K.J. (1993). The Hoffer Q formula: A comparison of theoretic and regression formulas. J. Cataract Refract. Surg..

[19] Barrett G.D. (1993). An improved universal theoretical formula for intraocular lens power prediction. J. Cataract Refract. Surg..

[20] Connell B.J., Kane J.X. (2019). Comparison of the Kane formula with existing formulas for intraocular lens power selection. BMJ Open Ophthalmol..

[21] Mencucci R., Cennamo M., Venturi D., Vignapiano R., Favuzza E. (2020). Visual outcome, optical quality, and patient satisfaction with a new monofocal IOL, enhanced for intermediate vision: Preliminary results. J. Cataract Refract. Surg..

[22] Tognetto D., Cecchini P., Giglio R., Turco G. (2020). Surface profiles of new-generation IOLs with improved intermediate vision. J. Cataract Refract. Surg..

[23] Rizzo S., Gambini G., De Vico U., Rizzo C., Kilian R. (2022). A One-Week Course of Levofloxacin/Dexamethasone Eye Drops: A Review on a New Approach in Managing Patients After Cataract Surgery. Ophthalmol. Ther..

[24] Rahman R., Stephenson J. (2014). Early surgery for epiretinal membrane preserves more vision for patients. Eye.

[25] Elliott D.B., Hotchkiss J., Scally A.J., Foster R., Buckley J.G. (2016). Intermediate addition multifocals provide safe stair ambulation with adequate “short-term” reading. Ophthalmic Physiol. Opt..

[26] (2017). Office for National Statistics Leisure Time in the UK: 2015. https://www.ons.gov.uk/economy/nationalaccounts/satelliteaccounts/articles/leisuretimeintheuk/2015.

[27] Bureau of Labor Statistics (2018). American Time Use Survey—2017 Results.

[28] Duan X.R., Liang Y.B., Friedman D.S., Sun L.P., Wei W.B., Wang J.J., Wang G.L., Liu W., Tao Q.S., Wang N.L. (2009). Prevalence and associations of epiretinal membranes in a rural Chinese adult population: The Handan Eye Study. Invest. Ophthalmol. Vis. Sci..

[29] Meuer S.M., Myers C.E., Klein B.E.K., Swift M.K., Huang Y., Gangaputra S., Pak J.W., Danis R.P., Klein R. (2015). The epidemiology of vitreoretinal interface abnormalities as detected by spectral-domain optical coherence tomography: The Beaver Dam Eye study. Ophthalmology.

[30] Klein R., Klein B.E.K., Wang Q., Moss S.E., Irvine A., Taylor H., Drews R., Crawford J.B., Schwartz A., Green W.R. (1994). The epidemiology of epiretinal membranes. Trans. Am. Ophthalmol. Soc..

[31] Auffarth G.U., Gerl M., Tsai L., Janakiraman D.P., Jackson B., Alarcon A., Dick H.B. (2021). Clinical evaluation of a new monofocal IOL with enhanced intermediate function in patients with cataract. J. Cataract Refract. Surg..

[32] Alarcon A., Cánovas C., Koopman B., Weeber H., Auffarth G.U., Piers P.A. (2020). Enhancing the Intermediate Vision of Monofocal Intraocular Lenses Using a Higher Order Aspheric Optic. J. Refract. Surg..

[33] Lee C.S., Gibbons L.E., Lee A.Y., Yanagihara R.T., Blazes M.S., Lee M.L., Mccurry S.M., Bowen J.D., Mccormick W.C., Crane P.K. (2022). Association Between Cataract Extraction and Development of Dementia. JAMA Intern. Med..

